# Bryophyte-Bioinspired Nanoporous AAO/C/MgO Composite for Enhanced CO_2_ Capture: The Role of MgO

**DOI:** 10.3390/nano14080658

**Published:** 2024-04-10

**Authors:** Paulina Jaqueline Cortés-Valadez, Esperanza Baños-López, Yazmín Mariela Hernández-Rodríguez, Oscar Eduardo Cigarroa-Mayorga

**Affiliations:** 1 Department of Advanced Technologies, UPIITA—Instituto Politécnico Nacional, Av. IPN 2580, Mexico City C.P. 07340, Mexico; pcortesv1700@alumno.ipn.mx; 2 Academia de Química, Universidad Autónoma del Estado de Hidalgo (UAEH), Carretera Pachuca-Tulancingo Km. 4.5., Pachuca C.P. 42184, Mexico; esperanza_banos10303@uaeh.edu.mx

**Keywords:** CO_2_ capture efficiency, alumina template, hydrothermal synthesis, MgO XPS, magnesium oxide nanostructures

## Abstract

A composite material composed of anodized aluminum oxide (AAO), carbon (C), and magnesium oxide (MgO) was developed for CO_2_ capture applications. Inspired by the bryophyte organism, the AAO/C/MgO composite mirrors two primary features of these species—(1) morphological characteristics and (2) elemental composition—specifically carbon, oxygen, and magnesium. The synthesis process involved two sequential steps: electroanodization of aluminum foil followed by a hydrothermal method using a mixture of glucose and magnesium chloride (MgCl_2_). The concentration of MgCl_2_ was systematically varied as the sole experimental variable across five levels—1 mM, 2 mM, 3 mM, 4 mM, and 5 mM—to investigate the impact of MgO formation on the samples’ chemical and physical properties, and consequently, their CO_2_ capture efficiency. Thus, scanning electron microscopy analysis revealed the AAO substrate’s porous structure, with pore diameters measuring 250 ± 30 nm. The growth of MgO on the AAO substrate resulted in spherical structures, whose diameter expanded from 15 nm ± 3 nm to 1000 nm ± 250 nm with increasing MgCl_2_ concentration from the minor to major concentrations explored, respectively. X-ray photoelectron spectroscopy (XPS) analysis indicated that carbon serves as a linking agent between AAO and MgO within the composite. Notably, the composite synthesized with a 4 mM MgCl_2_ concentration exhibited the highest CO_2_ capture efficiency, as determined by UV-Vis absorbance studies using a sodium carbonate solution as the CO_2_ source. This efficiency was quantified with a ‘k’ constant of 0.10531, significantly higher than those of other studied samples. The superior performance of the 4 mM MgCl_2_ sample in CO_2_ capture is likely due to the optimal density of MgO structures formed on the sample’s surface, enhancing its adsorptive capabilities as suggested by the XPS results.

## 1. Introduction

Alongside transitioning from fossil fuels to renewable energies like hydrogen, carbon recycling—utilizing CO_2_ as a resource—emerges as a viable strategy for achieving a carbon-neutral society [1]. Fujishima et al. introduced a method involving hydrogen extraction through water electrolysis using electricity from high-efficiency solar cells [2] (methodology currently used and highly studied [3]), followed by combining this hydrogen with CO_2_ emitted by power plants and factories to produce methanol, a potential energy source [4]. This process transforms carbon-containing gases like CO_2_ from greenhouse contributors into valuable resources, substituting oil and natural gas [5]. This concept harnesses the principles of artificial photosynthesis, which involves a nanostructured device designed to capture solar energy and convert CO_2_ emissions into fuel. Typically, an artificial photosynthesis device comprises complex materials that facilitate the energy conversion process [6]. Within the realm of artificial photosynthesis, a critical aspect is the development of materials geared towards the efficient physisorption of CO_2_. The focus is on synthesizing an active compound that can effectively capture CO_2_ through physical adsorption mechanisms [7]. 

Carbon dioxide (CO_2_) is identified as a significant greenhouse gas (GHG), contributing approximately 66% to radiative forcing due to long-lived GHGs [8,9]. Its presence in the atmosphere decreases the total loss of infrared radiation to space by absorbing and reflecting radiation emitted from the Earth’s surface, while oceans and ecosystems absorb the remainder [10]. The main causes include fossil fuel combustion, anthropogenic activities, coal energy, gasoline combustion, and population growth, leading to global warming [11]. The World Meteorological Organization reported atmospheric CO_2_ levels at 413.2 ppm ± 0.2 ppm in 2020, with atmospheric fractions showing significant variability over the past 60 years, ranging from 0.2% to 0.8% [12]. On the one hand, ocean absorption has led to an increase in sea surface temperatures and a decrease in pH due to CO_2_ uptake, which also slows down the meridional overturning circulation, contributing to the melting of sea ice [13]. Additionally, the deficiency in CO_2_ capture by nature is highlighted, attributed to the reduced ability of ecosystems and natural systems to adequately absorb and retain CO_2_ emissions from human activities such as fossil fuel combustion and deforestation [14,15]. Key factors behind this deficiency include deforestation and vegetation loss, land use changes, ocean acidification, wildfires and ecosystem degradation, atmospheric CO_2_ saturation, and changes in climate patterns [16]. These factors underscore the importance of addressing CO_2_ capture deficiency, adopting measures to protect and restore natural ecosystems, and reducing global CO_2_ emissions. Mitigating climate change and promoting sustainable practices are crucial for balancing the carbon cycle and limiting the adverse impacts of global warming [17]. 

In the context of escalating environmental challenges, notably the surge in greenhouse gases and climate change, bionics emerges as a promising field [18]. Defined by Lodato as the assimilation of engineering principles observed in natural systems and their application to the design or enhancement of technological systems or materials, bionics focuses on the technical transformation and application process to structures, methodologies, and principles of biological systems [19]. Biological systems are characterized by their sensitivity, high degree of flexibility, and adaptive function to various physical environments due to their high reliability. These features provide a vast research field to inspire biological systems for engineering applications, innovation, and solving day-to-day problems [20]. Particularly, bryophytes are represented by around 12,800 species worldwide, with 984 species and varieties recognized in Mexico [21]. The genus Sphagnum, commonly known as peat moss, was selected for this study. It includes between 150 to 350 different species. The specific species used in this work has not been identified, due to the need for comparison with species located in other countries. In this regard, this study focused on bryophytes from the moss genus, which absorb significantly more CO_2_ and water than other plants [22,23]. Carbon, oxygen, and magnesium (Mg) are elements that play a crucial role in CO_2_ adsorption and conversion into energy within green plants [24]. Furthermore, contemporary theoretical studies have suggested that the oxidation states of Mg significantly impact CO_2_ capture capabilities, with particular emphasis on the efficacy of the MgO phase in this process [25].

In this work, characterization techniques such as optical microscopy, SEM, FTIR, and XPS were utilized to investigate the morphology, physical properties, and chemical composition, aiming to replicate the key chemical and morphological features of bryophyte towards CO_2_ capture. Thus, anodized aluminum oxide (AAO) was synthesized utilizing the electroanodization technique, followed by employing a hydrothermal method in a glucose solution mixed with magnesium chloride (MgCl_2_) to fabricate the AAO/C/MgO composite. The concentration of MgCl_2_ was systematically varied to explore its impact on the Mg content’s oxidation states, as well as the physical and chemical properties, and ultimately, the CO_2_ capture efficiency. This research thoroughly examines the effect of MgCl_2_ concentration on the formation of the MgO phase and its correlation with CO_2_ capture efficiency. In addition, the relevance and novelty of this study are anchored in three pivotal areas: (1) the investigation of bryophyte’s chemical properties using advanced techniques such as X-ray photoelectron spectroscopy (XPS), (2) the detailed study of the AAO/C/MgO composite’s formation process, and (3) the comprehensive analysis of how the MgO phase influences the physical and chemical properties of the AAO/C/MgO composite, particularly in relation to CO_2_ capture efficiency. 

## 2. Materials and Methods

### 2.1. Materials and Reagents

Aluminum foil (Al, product number: 326860-3.6G), glucose (C_6_H_12_O_6_, product number: PHR1000-1G), magnesium chloride (MgCl_2_, product number 449172-10G), oxalic acid (C_2_H_2_O_4_, product number: 247537-500G), perchloric acid (HClO_4_, product number: 311421-250ML), phosphoric acid (H_3_PO_4_, product number: 695017-500ML), sodium hydroxide (NaOH, product number: 306576-100G), and deionized water (H_2_O, product number: W4502-1L with a resistivity of 18 MΩ) were procured from Sigma-Aldrich (St. Louis, MI, USA). These reagents were employed as provided, without any further purification, ensuring the consistency of experimental conditions.

### 2.2. Bryophyte Study for Bioinspired Elements Selection

A sample of 0.2 mg of bryophyte was subjected to comprehensive characterization through optical microscopy, scanning electron microscopy (SEM), Fourier-transform infrared spectroscopy (FTIR), and X-ray photoelectron spectroscopy (XPS) to elucidate its morphological, physical, and chemical attributes that contribute to bryophytes’ well-documented CO_2_ capture capabilities [23]. Prior to these analyses, the bryophyte sample was dehydrated using a 10% formalin solution for 24 h and subsequently dried under a nitrogen flow to prepare it for examination. The findings, as detailed later, identified two key characteristics that informed this research: (1) the high density of pores and (2) the presence of carbon, oxygen, and magnesium in the bryophyte’s chemical composition. These critical attributes, derived from the bryophyte’s characterization, underpin the design of the AAO/C/MgO composite, positioning it as a material inspired by natural processes [19]. This bioinspired approach leverages the inherent properties of bryophytes to enhance CO_2_ capture efficiency, reflecting a novel integration of natural mechanisms into material science. 

### 2.3. Synthesis of the Nanoporous AAO/C/MgO

The anodized aluminum oxide (AAO) substrate was synthesized using the established two-step anodization method [26,27]. Briefly, aluminum foil was cut into 10 mm diameter circles and underwent mechanical polishing to achieve a mirror finish. Electropolishing followed, utilizing a potentiostat in a 4:1 mixture of perchloric acid and ethanol, applying a steady 20 V for one minute at room temperature. Residual acid was thoroughly removed via four ultrasonic cleaning cycles in deionized water and methanol, with subsequent nitrogen drying. The substrate was then anodized in 3 M oxalic acid at 20 V for two hours at 4 °C to form the initial alumina layer. This layer was dissolved in 0.5 M phosphoric acid for 20 min, succeeded by a second anodization for six hours to refine the pore structure. A final cleaning in six ultrasonic cycles and nitrogen drying completed the process, yielding an alumina structure with enlarged pores and increased surface area. An aqueous solution of 10 mM glucose was prepared and combined with varying concentrations of MgCl_2_ aqueous solution. The mixture was subjected to magnetic stirring for 30 min to ensure thorough mixing. MgCl_2_ concentrations of 1 mM, 2 mM, 3 mM, 4 mM, and 5 mM were investigated, keeping the glucose concentration constant at 10 mM. To facilitate the formation of the C/MgO complex, the established hydrothermal method was utilized [28]. Thus, the mixture was transferred into a cylindrical stainless-steel vial, equipped with a Teflon liner to prevent sample contamination from reactions with the stainless-steel interior. Additionally, 0.2 mg of AAO was added to the mixture to aid in forming the AAO/C/MgO complex. The system was then heated to 180 °C for 4 h to conduct hydrothermal synthesis. Post synthesis, the samples were cleaned with two ultrasonic baths in deionized water and dried under a nitrogen flow. To ensure reproducibility, the samples were synthesized in triplicate across separate experiments. Please note that the choice of 4 °C for the synthesis of AAO was deliberately made to achieve small pores within the alumina substrates. This is because, as documented in the literature [26], higher temperatures tend to result in larger pore diameters, whereas lower temperatures favor the formation of smaller pores. This principle is well established in the anodization method for alumina synthesis from aluminum, underscoring the critical role of temperature control in tailoring the pore size to desired specifications.

### 2.4. Physical and Chemical Characterization

The samples underwent a comprehensive characterization process. Initially, optical microscopy was employed for initial visualization. The vibrational modes were thoroughly analyzed using Fourier-transform infrared spectroscopy (FTIR), where samples mixed with potassium bromide (KBr) were subjected to a temperature of 150 °C for 24 h. High-resolution measurements were carried out with a Varian 660-IR spectrometer (Agilent, Santa Clara, CA, USA), set to 1 cm^−1^ resolution, and comprising 20 scans per measurement to guarantee the accuracy and reliability of the spectral data. For morphological analysis, field-emission scanning electron microscopy (FE-SEM) was performed using a JEOL 7401F microscope (Tokyo, Japan), enabling detailed observation of the samples’ surface and structure. Additionally, chemical composition and atomic-level studies were conducted using X-ray photoelectron spectroscopy (XPS) on a Thermo Fisher Scientific K-alpha model (Waltham, MA, USA). This utilized a monochromatic Alkα X-ray source for excitation, ensuring precise compositional analysis. All measurements were executed at room temperature to ensure consistent and reliable conditions across the analyses.

### 2.5. Evaluation of CO_2_ Capture 

The CO_2_ capture efficiency of AAO/C/MgO nanostructures was rigorously evaluated following the methodology outlined by Mendoza-Sánchez et al. [7]. This involved exposing the samples to CO_2_ generated from the reaction of sodium carbonate (Na_2_CO_3_) with hydrochloric acid (HCl), yielding sodium chloride (NaCl), water (H_2_O), and carbon dioxide (CO_2_). The samples’ interaction with CO_2_ was conducted within a hermetically sealed quartz cell, engineered to specifically assess their CO_2_ adsorption potential. A 186 mM sodium carbonate solution was used for these tests. CO_2_ adsorption was indirectly quantified by observing changes in the absorbance of the solution over time, employing a UV–Vis spectrophotometer (i3 UV-VIS SPECTROPHOTOMETER, Hanon Instruments, Shanghai, China) at room temperature. Absorbance spectra were captured every 15 min over a span of 180 min to ensure comprehensive data collection.

## 3. Results and Discussion

### 3.1. Bryophyte Characterization

**Figure 1:**
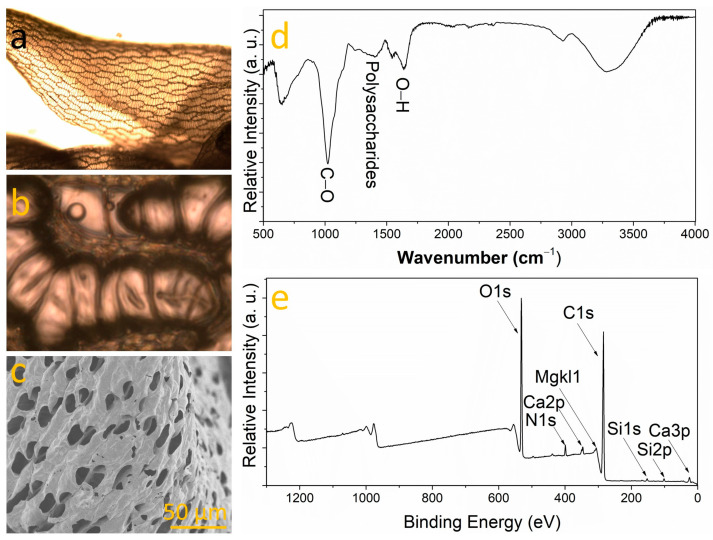
(**a**) Optical microscope image of bryophyte, (**b**) zoomed area, (**c**) FESEM close-up. (**d**) FTIR and (**e**) XPS spectra of bryophyte.

Optical microscopy revealed a heterogeneous appearance with dark brown areas indicating potential decomposition or water stress, alongside green zones suggestive of ongoing photosynthetic activity (Figure 1a). This diversity in color and texture could denote various growth stages or environmental stress responses, particularly as the sample underwent drying for integration purposes [29]. Such features are typical of bryophytes, non-vascular plants renowned for their resilience in moist environments and their pivotal role in ecosystems, contributing to soil formation and serving as environmental quality indicators. Microscopic analysis unveiled a net-like structure composed of predominantly hexagonal, polygonal cells outlined by dark lines, possibly representing cell walls, and featuring a light, uniform color across the sample. A detailed examination (Figure 1b) highlighted smaller, more compactly organized structures with grayish-brown coloration. Additionally, it showed a mesh-like structure with irregularly sized and shaped polygonal cells, where the demarcating lines were thicker and darker, and the cell interiors varied from light to dark brown, implying a more textured or three-dimensional aspect [30]. Particularly noteworthy are the larger, lighter hyaline cells unique to Sphagnum (bryophyte), essential for water retention [31]. Chloroplasts, vital for photosynthesis, were observed in the smaller green cells, termed chlorophyllic cells. The elongated cells, arranged in a regular pattern with significant intercellular spaces, are likely hyaline cells specializing in water retention, as illustrated in Figure 1b. Figure 1c shows an FESEM image of a Sphagnum surface. It highlights the openings or pores on the surface of the hyaline cells, characteristic of Sphagnum. The fibers or hanging structures could be remnants of cells or structural elements of the moss. Therefore, the high density of pores observed in bryophytes may serve as a critical morphological feature for CO_2_ adsorption, as it enhances the physical accessibility of CO_2_ molecules to the active sites involved in the capture process. The FTIR spectrum is shown in Figure 1d, where the band at 989 cm^−1^ is attributed to cellulose C-O stretching, the band at 1013 cm^−1^ to pectin groups, the band at 1126 cm^−1^ to polysaccharides, and the band at 3321 cm^−1^ to cellulose O-H bending [32,33,34,35]. This suggests a significant presence of organic molecules in the sample as expected. On the other hand, the XPS analysis, as illustrated in Figure 1e, revealed the elemental composition of the sample, identifying the presence of oxygen, carbon, calcium, nitrogen, silicon, and magnesium. This was determined through the respective energy binding (EB) bands for each element: O1s, C1s, Ca2p, N1s, Si2p, and MgKl1 [36,37,38,39,40]. The analytical results reveal both the atomic percentage and the weight percentage of elements detected in the sample: 68.1% of the atoms in the sample are carbon (C1s), followed by oxygen (26.7%) and nitrogen (2.9%). Minor quantities of silicon (1.1%), calcium (0.8%), sulfur (0.2%), sodium (0.2%), and magnesium (0.2%) were also detected. Overall, the sample predominantly consists of carbon and oxygen, with only a minor fraction of atoms belonging to elements other than C, O, and N, typically attributed to plant samples [41]. Consequently, calcium (Ca) and magnesium (Mg) might be integral to the active components within the structures facilitating CO_2_ absorption in the bryophyte. Two primary factors appear to enhance the bryophyte’s CO_2_ capture efficiency: (1) its substantial porosity and (2) its chemical composition. Inspired by these findings, a strategy was adopted to utilize a porous substrate and investigate the role of magnesium in synthetic samples, aiming to analyze its impact on CO_2_ capture efficiency.

### 3.2. Influence of MgCl_2_ on the Morphology of MgO on AAO

**Figure 2:**
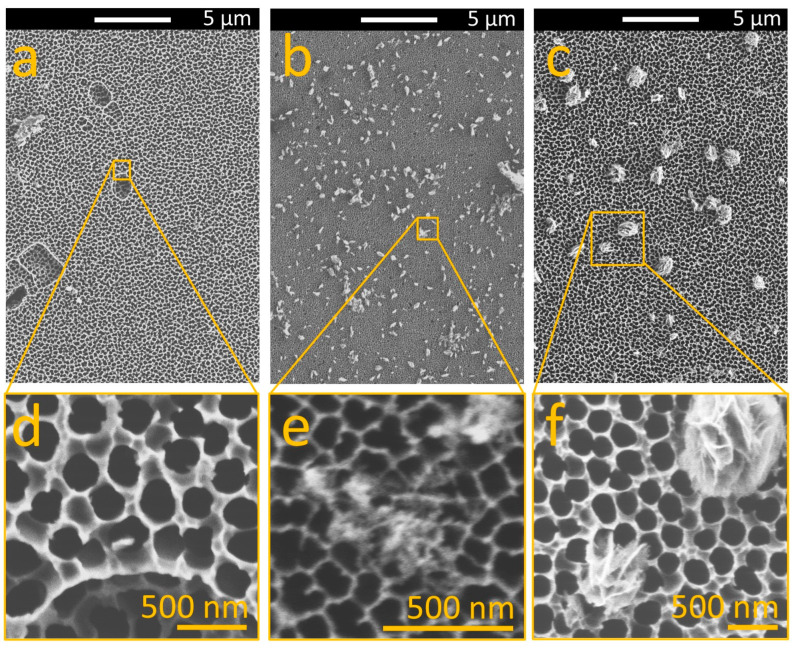
FESEM image of front view of (**a**) pristine AAO, (**b**) AAO post-C-MgO integration with 1 mg MgCl_2_, and (**c**) AAO post-C-MgO integration with 5 mg MgCl_2_. Images (**d**–**f**) provide detailed close-ups of (**a**), (**b**), and (**c**), respectively.

Once the AAO/C/MgO complex was synthesized, it was characterized by FESEM to understand the achieved morphology across MgCl_2_ variations. Figure 2a shows the AAO substrate as obtained. As can be seen, the porosity on the sample is extended across the entire surface of the sample with regular size. After the hydrothermal synthesis was complete, small structures were attached to the entire surface of the sample. Figure 2b shows the sample obtained after the hydrothermal synthesis with a 1 mM solution of MgCl_2_. On the other hand, Figure 2c shows the AAO after the hydrothermal synthesis employing the aqueous solution of MgCl_2_ with a concentration of 5 mM, where the presence of larger structures on the entire surface of the AAO can be seen. A close-up of the samples allows the comparison of the AAO before and after the hydrothermal process. Figure 2d shows the pores in the AAO (black circles). The pores have well-aligned tubular pores (which is in good agreement with the AAO obtained by the anodization method [42]) with a diameter of 250 ± 30 nm (measured by FESEM analysis). Please note that AAO is a well-established material, renowned for its characteristic formation of well-aligned cylindrical pores [26,27]. After the hydrothermal synthesis, the sample obtained with the minor MgCl_2_ concentration that was explored (1 mM) depicts a relatively smooth surface sprinkled with particles of varying sizes. These particles exhibit an irregular morphology and are randomly distributed across the surface. These particles comprise small nanoparticles (diameter 15 nm ± 3 nm) of a combined phase of C and MgO present on the entire alumina surface (see Figure 2e). Once the MgCl_2_ concentration is increased in the hydrothermal synthesis, the particles increase in size and form flakes until the formation of microparticles (diameter of 1000 nm ± 250 nm) attached to the surface of the AAO when the maximum explored MgCl_2_ concentration (5 mM) is employed (see Figure 2f). Thus, the particles now exhibit an elongated and fibrous morphology, standing out against the AAO substrate. Please note that the appearance of the AAO in Figure 2b,e might seem distinct from that in Figure 2a,c,d,f. This discrepancy is attributed to charge effects encountered during FESEM (Field Emission Scanning Electron Microscopy) analysis, as AAO (anodized aluminum oxide) is an electrically insulating material. Analyzing such materials with electron microscopy techniques poses a significant technical challenge due to these charge effects. However, this does not indicate that the pore diameter of the AAO is altered during the synthesis process. The stability of alumina is well documented to remain consistent across various temperatures and chemical reactions, ensuring the integrity of the AAO structure throughout our study. In addition, the MgCl_2_ has a direct influence on the morphology of the AAO/C/MgO composite. Thus, at lower concentrations of MgCl_2_, the MgO structures formed on the AAO are typically smaller and more uniformly distributed. As the concentration of MgCl_2_ increases, the MgO structures begin to grow in size and change shape.

**Figure 3:**
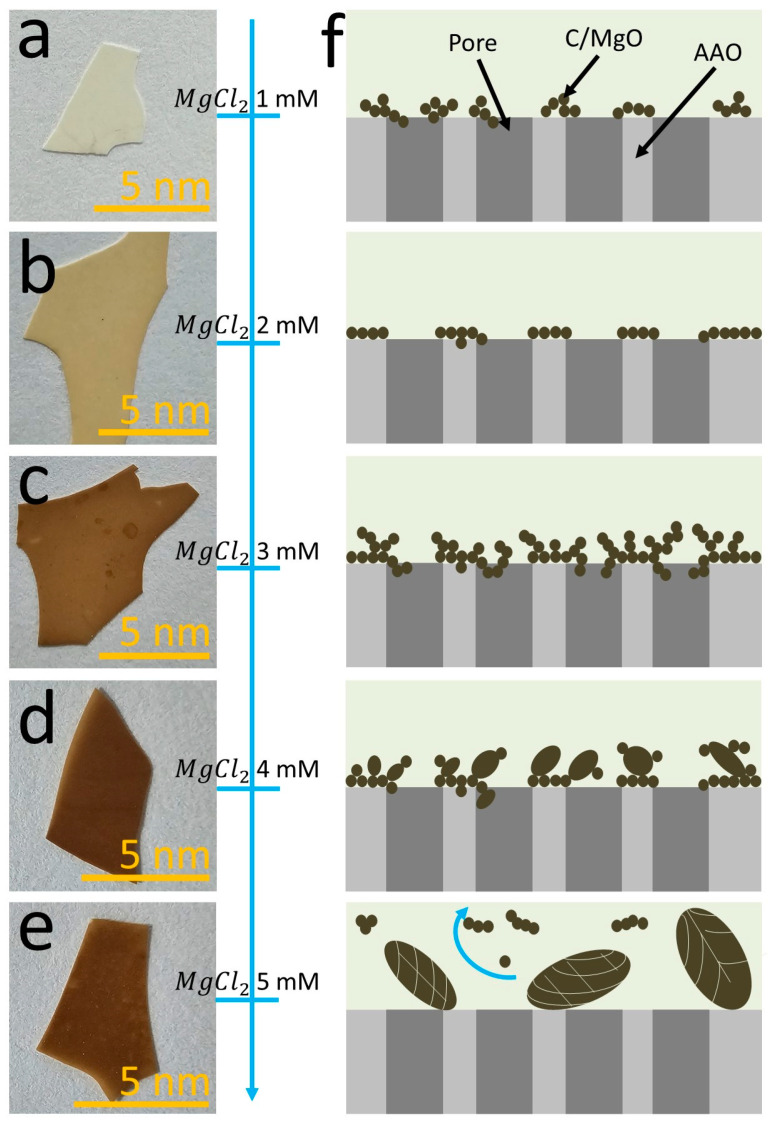
Optical microscope images showcase AAO/C/MgO synthesized with varying MgCl_2_ concentrations: (**a**) 1 mM, (**b**) 2 mM, (**c**) 3 mM, (**d**) 4 mM, and (**e**) 5 mM. Image (**f**) provides a schematic lateral view of the samples, illustrating changes across the MgCl_2_ gradient in the synthesis process. Please note that the blue arrow shows the explored MgCl_2_ concentration.

Optical images of the top view of the AAO/C/MgO complex synthesized with 1 mM, 2 mM, 3 mM, 4 mM, and 5 mM solutions of MgCl_2_ are shown in Figure 3a, Figure 3b, Figure 3c, Figure 3d, and Figure 3e, respectively. The initial formation starts at lower MgCl_2_ concentrations (1 mM), and the hydrothermal synthesis initiates the deposition of C-MgO complexes on the AAO surface. This process results in the formation of small, irregularly shaped particles that are randomly distributed across the AAO substrate. These initial formations are characterized by their nanoparticle size (approximately 15 nm ± 3 nm), suggesting that the low MgCl_2_ concentration facilitates the nucleation of discrete C-MgO composite particles without significantly altering the inherent porosity of the AAO substrate. For the 2 mM of MgCl_2_ solution, a regular end uniform cover of the C/MgO complex is deposited on the AAO substrate. Then, as the concentration of MgCl_2_ is increased in the hydrothermal process, there is a notable transition in the morphology of the deposited structures. The particles begin to increase in size and adopt larger flat-like structures with morphology. This change indicates that higher MgCl_2_ concentrations promote the aggregation of C-MgO complexes, leading to the formation of larger structures while still maintaining the distribution of these formations across the porous AAO surface. At the highest investigated MgCl_2_ concentrations (5 mM), the hydrothermal synthesis process culminates in the formation of microparticles with diameters of approximately 1000 nm ± 250 nm. These larger structures exhibit an elongated and fibrous morphology, significantly distinct from the smaller particles formed at lower concentrations. This advanced stage of formation suggests that the increased MgCl_2_ concentration facilitates the growth and coalescence of C-MgO complexes into larger microparticles. Figure 3f shows a scheme that represents the growing formation of the AAO/C/MgCl_2_ complex. In addition, the increase in MgCl_2_ concentration during the hydrothermal procedure systematically influences the morphology of the C-MgO structures formed on the AAO substrate. 

### 3.3. Chemical Nature of Bindings in AAO/C/MgO

**Figure 4:**
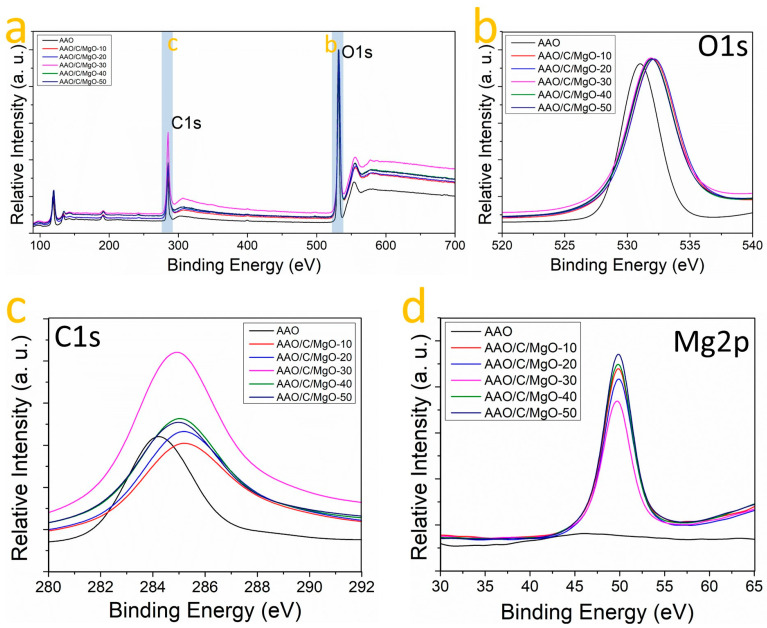
XPS analysis of samples: (**a**) overall survey; close-ups of (**b**) 520–540 eV, (**c**) 280–292 eV, and (**d**) 290–340 eV ranges.

To corroborate the chemical composition of the AAO/C/MgO composite previously proposed, XPS spectra analysis was used to identify the elements present in the samples. Figure 4a shows the comparison of XPS survey spectra obtained from the samples, revealing the presence of key elements such as aluminum, magnesium, carbon, and oxygen. Carbon and oxygen are highlighted in Figure 1a, where peaks corresponding to the C1s binding energy (BE) and the O1s BE bands are clearly identified, indicating the successful incorporation of these elements into the samples [43,44]. In Figure 4b, the O1s BE band region from all the investigated samples is presented, showing a noticeable shift towards higher energies in the spectra following the C/MgO incorporation into the AAO substrate. This shift suggests an increase in chemisorbed oxygen within the samples [26,45], implying that oxygen atoms play a significant role in the interaction with the C/MgO complex on the AAO support. Such an increase in chemisorbed oxygen could enhance the materials’ properties, particularly in applications where oxygen reactivity is crucial. Further analysis in Figure 4c compares the C1s BE band across the samples, where a distinct shift of 0.75 eV towards higher energies is observed post-hydrothermal treatment for the AAO/C/MgO complex formation. This shift indicates a transformation in the hybridization state of carbon atoms from sp2 to sp3 following the hydrothermal process [46]. This transformation corroborates the critical role of carbon in facilitating the linkage between AAO and MgO, suggesting modifications in the electronic structure and bonding environment of carbon that could influence the overall functionality of the synthesized material. Additionally, Figure 4d presents the Mg2p BE band comparison. Notably, the Mg2p signal is absent in untreated AAO samples, whereas in post-hydrothermal treatment, the signal emerges for all samples. 

**Figure 5:**
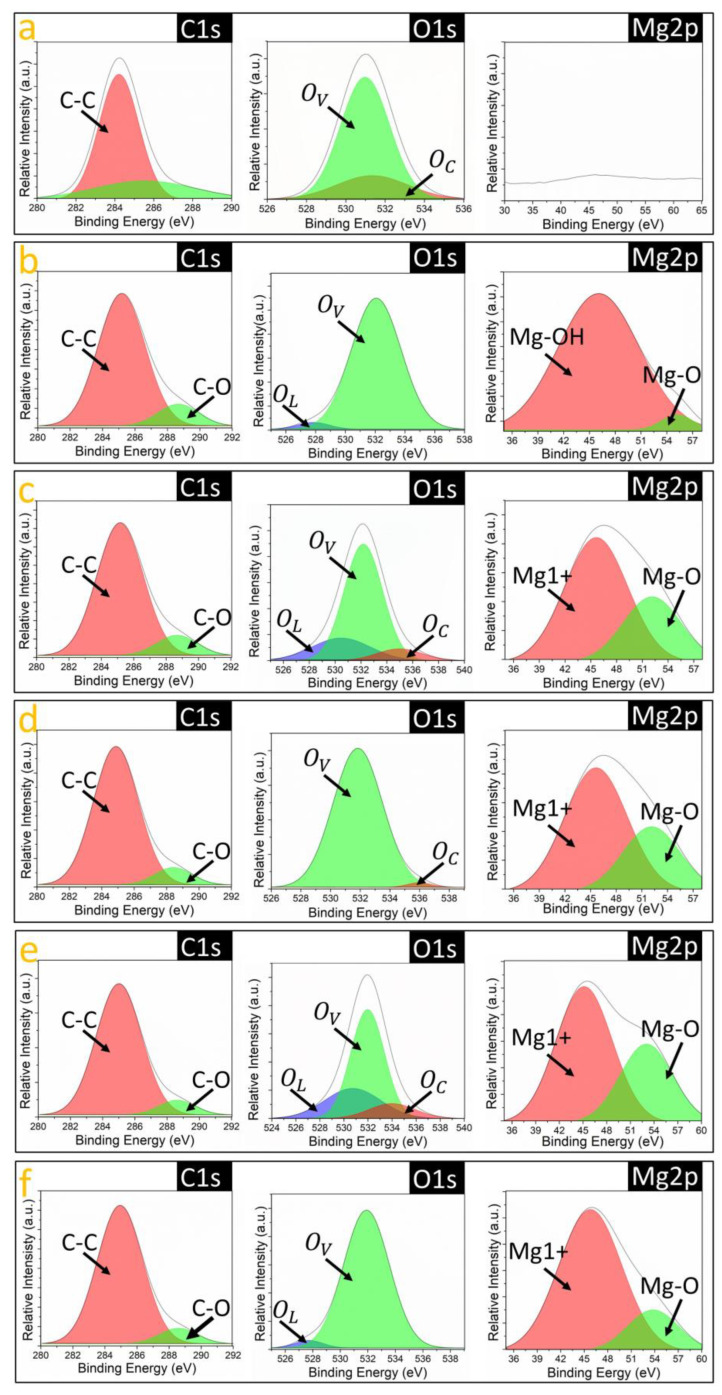
Gaussian fitting of the XPS spectra for O1s BE, C1s BE, and Mg2p BE bands of the samples (**a**) AAO, (**b**) AAO/C/MgO-10, (**c**) AAO/C/MgO-20, (**d**) AAO/C/MgO-30, (**e**) AAO/C/MgO-40, and (**f**) AAO/C/MgO-50.

The presence of the Mg2p EB band is indicative of the MgO phase [47], highlighting the successful integration of MgO into the AAO framework. The emergence of this signal further supports the formation of the AAO/C/MgO complex and suggests that the hydrothermal process effectively induces the incorporation of magnesium into the structure. The XPS data underwent Gaussian fitting to elucidate the oxidation states of Mg, O, and C more clearly, as shown in Figure 5. For the C1s BE band, two main components were identified (refer to Figure 5a). In the case of the AAO sample, a component located at 284.5 eV corresponds to C-C bonding [48], while the other component was identified as a background signal, likely resulting from fluorescence [49]. Following the hydrothermal procedure (Figure 5b–f), a second component emerging at 288.7 eV was observed, which is attributed to C-O bonding [48]. This finding suggests the involvement of carbon atoms in linking the AAO substrate with the MgO phase. On the other hand, the O1s BE band is composed of the oxygen species present in the sample. These species include oxygen vacancies (O_V_), chemisorbed oxygen (O_C_), and lattice oxygen vacancies (O_L_) [50]. Applying a Gaussian fit to the O1s EB data within this study has enabled the detailed identification of O_V_, O_C_, and O_L_ species. The O1s binding energy (BE) band, depicted in Figure 5a, reveals the presence of oxygen vacancies (O_V_) and chemisorbed oxygen (O_C_) within the sample, as indicated by the component peaks at 530.93 eV and 531.35 eV, respectively. Following the hydrothermal treatment, a notable shift in the intensity ratios of these components is observed (Figure 5b–f), indicating a change in the oxygen species composition within the samples. Specifically, the samples synthesized with 2 mM and 4 mM concentrations of MgCl_2_ demonstrated the presence of all three oxygen-related components, including lattice oxygen vacancies (O_L_), which is particularly significant. Particularly, the O_L_ EB is indicative of oxygen atoms bonded to Mn or Fe family metals such as Mn, which reinforces the idea about the presence of the MgO phase [51]. Finally, the Mg2p component was subjected to Gaussian fitting to analyze the Mg oxidation states associated with the samples. As anticipated, the AAO sample did not display an Mg2p binding energy (BE) band (see Figure 5a), which is consistent with the absence of MgCl_2_ addition during its preparation. Conversely, following the C/MgO incorporation process, two distinct components were identified within the Mg2p BE band, located at approximately 44.8 eV and 53.6 eV (see Figure 5b–f). These components have been attributed to the Mg1+ species and Mg-O bonding, respectively [52]. In addition, the detection of the Mg-O-related signal confirms the formation of the MgO phase, while the signal associated with the Mg1+ species indicates that Mg predominantly contributes to the linkage of C to the MgO structures adhered to the AAO surface. Notably, the sample synthesized with a 4 mM MgCl_2_ concentration exhibited the highest Mg-O-to-Mg1+ ratio (as shown in Figure 5e), implying that this sample possesses the greatest concentration of MgO structures available on the AAO surface. 

### 3.4. CO_2_ Adsorption of the AAO/C/MgO

**Figure 6:**
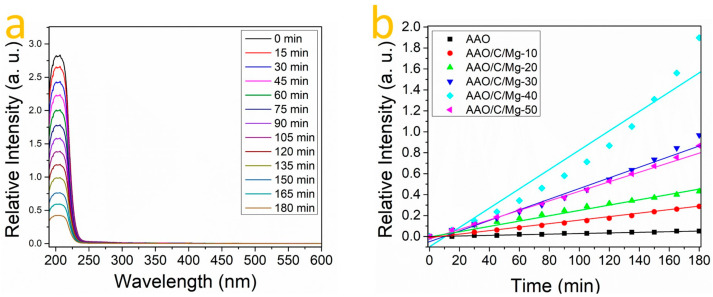
UV-VIS absorbance spectra for Na_2_CO_3_ dissolution with (**a**) AAO/C/MgO synthesized with MgCl_2_ 4 mM over time. (**b**) Kinetic comparison of CO_2_ adsorption between samples.

Figure 6a displays a series of absorbance curves for the sample labeled AAO/C/MgO-40 during a 180 min exposure to CO_2_, as detailed in Section 2.4 of the experimental procedure. The curves exhibit a progressive decrease in absorbance over time, which is indicative of the sample’s CO_2_ absorption on its surface. This diminishing trend suggests that as the CO_2_ interacts with the surface of the sample, it is steadily captured, thereby reducing the amount of light absorbed at specific wavelengths—this being a direct measure of the CO_2_ concentration around the sample. The same procedure was meticulously applied to all samples to construct a comprehensive kinetic profile of CO_2_ interaction with the various synthesized materials. By comparing the rate and extent of absorbance reduction across different samples, the relative efficiencies of CO_2_ capture can be ascertained. This comparative analysis is crucial for understanding the dynamics of CO_2_ adsorption and for identifying which samples exhibit the most promising characteristics for effective CO_2_ sequestration. The kinetics derived from these experiments are essential for modeling the adsorption process and for scaling up the technology for practical CO_2_ capture applications. Figure 6b illustrates the CO_2_ capture kinetics for different samples. The x-axis represents time in minutes, while the y-axis shows the relative intensity, which might correlate with the amount of CO_2_ absorbed. Each line represents a sample treated with different concentrations of MgCl_2_, ranging from 1 mM to 5 mM, labeled as AAO/C/Mg-10 to AAO/C/Mg-50, respectively, with AAO serving as a control. The upward trend of the lines suggests that CO_2_ capture increases over time. The steeper the slope, the more efficient the CO_2_ capture. It is evident that samples treated with higher MgCl_2_ concentrations (AAO/C/Mg-50, for example) demonstrate a more pronounced increase in relative intensity over time, indicating a potentially higher CO_2_ capture efficiency compared to those with lower MgCl_2_ concentrations (AAO/C/Mg-10). Based on the literature, the k constant was computed [53]. The rate constant ‘k’ for CO_2_ capture was meticulously computed, revealing a notable dependence on the magnesium content within the samples, as detailed in Table 1. The tabulated data underscore the direct correlation between Mg concentration and CO_2_ capture efficacy, with varying degrees of influence observed across the range of samples tested. From the values reported in Table 1, it is evident that the Mg content plays a pivotal role in the adsorption process. The AAO/C/MgO-40 sample, synthesized using a 4 mM MgCl_2_ solution, exhibited the highest ‘k’ constant of 0.10531, significantly outperforming its counterparts. This result positions the AAO/C/MgO-40 composite as the most proficient in CO_2_ sequestration, suggesting that this specific MgCl_2_ concentration provides an optimal balance that enhances the adsorptive interactions between CO_2_ and the material’s surface. By contrast, the pristine AAO sample, devoid of MgO integration, registered the lowest ‘k’ constant at a mere 0.00028, underscoring the dramatic effect of C/MgO incorporation on CO_2_ capture capabilities. A gradual increase in efficiency is observed with the addition of MgCl_2_, as evidenced by the samples AAO/C/MgO-10 and AAO/C/MgO-20, which show ‘k’ constants of 0.00159 and 0.00239, respectively. However, upon further incrementing the MgCl_2_ concentration beyond the optimal level to 50 mM, as in the AAO/C/MgO-50 sample, the ‘k’ constant decreases to 0.00481. This suggests a diminishing return on efficiency, potentially due to an over-concentration of Mg leading to a detrimental aggregation effect or a decrease in available active sites for CO_2_ binding. The K constant displayed by the sample AAO/C/MgO-40 is approximately 376 times greater than that of pure AAO, positioning it as the most efficient material for CO_2_ capture among all the samples analyzed. This significant enhancement in CO_2_ capture efficiency can be attributed to two primary factors: (a) the quantity of MgO present on the surface of the samples, which increases the availability of active sites for CO_2_ adsorption, and (b) the distinct morphology of the MgO structures, which is directly influenced by variations in MgCl_2_ concentration according to the presented results. 

A notable trend was observed in the hydrothermal synthesis process: when utilizing a solution with a concentration higher than 4 mM MgCl_2_, the CO_2_ capture efficiency unexpectedly decreased. This reduction in efficiency could be due to a loss of material from the substrate surface, suggesting that the optimal threshold for a homogeneous coating on the AAO surface is achieved at this particular MgCl_2_ concentration. Beyond this point, the additional material may not adhere as effectively, leading to a potential shedding or blocking of active sites crucial for CO_2_ adsorption. The supposition is supported by optical observations detailed in Figure 3. The images likely show that at concentrations higher than 4 mM, there is a visible change in the surface morphology, indicating an oversaturation that does not contribute to—or may even detract from—CO_2_ capture capabilities. It is plausible that the maximum capacity for adsorption is reached with the 4 mM solution, and any further addition of MgCl_2_ leads to a superfluous layer that hinders the accessibility of CO_2_ to the adsorptive sites. This observation is critical for understanding the material’s adsorption dynamics and for optimizing the synthesis protocol for AAO/C/MgO composites intended for CO_2_ sequestration. It emphasizes the delicate balance between the quantity of active material and its distribution across the substrate, which is essential for maximizing CO_2_ capture efficiency. Future investigations could explore the precise mechanisms that cause the decline in capture efficiency beyond the 4 mM MgCl_2_ concentration, potentially leading to refined strategies for material synthesis in carbon capture applications.

MgO plays a pivotal role in enhancing CO_2_ capture efficiency, as evidenced by the comprehensive data and analysis provided in this work. This idea is highlighted by the strong efficiency in CO_2_ capture exhibited by the AAO/C/MgO-40 sample, the sample with the maximum availability of MgO structures on the surface of AAO. Thus, the incorporation of MgO on the anodized aluminum oxide (AAO) matrix, facilitated through a hydrothermal synthesis process involving magnesium chloride (MgCl_2_), significantly influences the composite’s physicochemical properties and its ability to adsorb CO_2_. The presence of MgO introduces additional active sites for CO_2_ adsorption, as indicated by the shift in binding energies observed in XPS analyses, attributed to its high surface reactivity and affinity for CO_2_ molecules. The optimization of MgCl_2_ concentration, particularly at 4 mM, results in an ideal distribution and morphology of MgO structures on the AAO surface, maximizing the composite’s CO_2_ capture capacity. This optimized MgO content not only enhances the physical accessibility of CO_2_ to the adsorption sites but also contributes to the formation of bonds between CO_2_ molecules and the composite surface. Therefore, MgO’s role in the AAO/C/MgO composite is critical for achieving high CO_2_ capture efficiency. Conversely, while porosity is recognized as a crucial factor for CO_2_ capture [54], this work did not extensively investigate this parameter, focusing instead on maximizing control over the primary variable of interest: MgCl_2_ concentration in the synthesis of the composite. Simultaneously, the expected values for porosity and surface area were anticipated to be comparable across all samples, given that the AAO substrate—which significantly contributes to both porosity and surface area—was synthesized under consistent conditions for each sample. This approach ensured a standardized baseline from which the influence of MgCl_2_ concentration on CO_2_ capture efficiency could be accurately assessed, without the variability in porosity and surface area confounding the results. Moreover, when comparing the CO_2_ capture efficiency achieved by the AAO/C/MgO-40 composite with other materials documented in the literature, it becomes evident that the results of this study are on par with, if not superior to, those of other materials, especially considering the minimal sample quantity used for the CO_2_ capture tests in this research. Table 2 presents a comparison of various materials recently synthesized and designed for CO_2_ capture, highlighting their capture efficiencies. Consequently, MgO emerges as a promising candidate for the development of materials tailored for CO_2_ capture applications, demonstrating its potential in significantly enhancing efficiency in this critical area. This comparative analysis further establishes the AAO/C/MgO-40 composite’s notable performance and positions MgO as a key component in the advancement of CO_2_ capture technology.

Despite the various materials reported in the literature with high CO_2_ capture efficiencies, this study introduces a pioneering investigation into the role of magnesium oxide (MgO) in enhancing CO_2_ adsorption within the AAO/C/MgO composite. Through the precise manipulation of magnesium chloride (MgCl_2_) concentration during the synthesis process, this research highlights the pivotal role of MgO in improving the composite’s CO_2_ adsorption efficiency. The discovery of an optimal MgCl_2_ concentration of 4 mM, which promotes the formation of MgO structures on the AAO surface with superior CO_2_ capture properties, illuminates the previously overlooked effects of MgO’s oxidation states and its interaction with carbon and aluminum oxide in the composite. This breakthrough provides valuable insights into the fundamental mechanisms by which MgO contributes to CO_2_ capture, opening new avenues for the development of more effective CO_2_ adsorption materials inspired in biological systems. The innovative findings emphasize the potential of bioinspired materials to address environmental challenges and represent a notable step forward in harnessing MgO for CO_2_ capture applications.

### 3.5. Future Prospects of the AAO/C/MgO Composite

To further enhance the CO_2_ capture efficiency of this already efficient composite in future research, the following recommendations are proposed:

Optimization of MgCl_2_ concentration: while the 4 mM MgCl_2_ concentration has proven to be effective, further fine-tuning this concentration within a narrower range could potentially identify a more precise optimal point that maximizes the MgO structure’s efficacy in CO_2_ adsorption.

Surface modification: Investigating surface treatments or modifications to the AAO/C/MgO composite that could increase the active surface area or introduce additional functional groups that facilitate CO_2_ adsorption. This could involve doping with other metals or non-metals that synergize with MgO’s adsorption properties.

Composite structure refinement: exploring variations in the hydrothermal synthesis parameters, such as temperature, duration, and the glucose-to-MgCl_2_ ratio, could lead to a more controlled growth of MgO structures, potentially yielding even more effective CO_2_ adsorbing materials.

Pore size optimization: since the efficiency of CO_2_ capture can also be influenced by the pore size of the AAO substrate, further studies could focus on optimizing the electroanodization conditions to produce AAO with tailored pore sizes that match the kinetic diameter of CO_2_ molecules more closely.

Hybrid composites: investigating the incorporation of other bioinspired materials or nanomaterials with the AAO/C/MgO composite could lead to hybrid materials that leverage multiple mechanisms for CO_2_ capture, potentially enhancing efficiency beyond the capabilities of single-component systems.

By focusing on these areas for further research, it is anticipated that the efficiency of the AAO/C/MgO composite for CO_2_ capture can be significantly improved, contributing to the development of more effective and sustainable solutions for addressing CO_2_ emissions.

## 4. Conclusions

The AAO/C/MgO complex was achieved by an accessible hydrothermal method. In addition, the rate constant ‘k’ revealed a pronounced effect of Mg content on capture efficiency. Our findings suggest that there is an optimal MgCl_2_ concentration in the synthesis process that maximizes CO_2_ capture efficiency. Specifically, the sample synthesized with a 4 mM MgCl_2_ solution exhibited the highest ‘k’ value at 0.10531, indicating superior CO_2_ sequestration compared to other concentrations. However, it is essential to note that beyond this optimal point, an increase in MgCl_2_ concentration to 5 mM resulted in a decrease in ‘k’ value to 0.00481, suggesting a threshold for the beneficial impact of Mg on the CO_2_ capturing process. FESEM characterization of the synthesized AAO/C/MgO composites further supported these findings. The samples treated with 4 mM MgCl_2_ solution showed homogeneous coverage on the AAO surface with well-aligned tubular nanopores, which is crucial for effective CO_2_ adsorption. The synthesis of AAO/C/MgO composites for CO_2_ capture is highly dependent on the precise concentration of MgCl_2_ used in the hydrothermal process. The optimization of magnesium chloride (MgCl_2_) concentration during the hydrothermal synthesis process revealed that a 4 mM concentration is optimal for creating MgO structures on the anodized aluminum oxide (AAO) surface, significantly enhancing CO_2_ adsorption capabilities. This optimal concentration fosters the development of MgO available on the AAO substrate surface. The knowledge gained from this study is invaluable for the future development of CO_2_ capture technologies, providing a foundation for optimizing material design and synthesis to combat the increasing levels of CO_2_ in the atmosphere.

## Figures and Tables

**Table 1:** Comparison between k constants computed at 180 min.

Sample	K Constant
AAO	0.00028
AAO/C/MgO-10	0.00159
AAO/C/MgO-20	0.00239
AAO/C/MgO-30	0.00537
AAO/C/MgO-40	0.10531
AAO/C/MgO-50	0.00481

**Table 2:** Comparison of CO_2_ capture efficiency achieved by the sample AAO/C/MgO-40 against other materials reported in the literature.

Material	Amount of CO_2_ Adsorption	Time	Reference
ZIF-8-W/[TEPA][MIm]	2.22 mol/mol ILs	65 min	[55]
Ti2C-MXene/activated carbon nanocomposite	67.83 cm^3^/g	6 h	[56]
Amine-grafted on boron-modified SBA-15	0.79 mmol/g	300 min	[57]
Tetraethylenepentamine-modified Cu_2_(OH)PO_4_	0.67 molCO_2_/molN	22 min	[58]
Ultramicroporous carbon microspheres	0.24 cm^3^/g	6 h	[59]
AAO/C/MgO-40	1.66 mmol/g	180 min	Reported in this work

## Data Availability

All data used in this research are available upon request.

## References

[1] dos Reis E.A., da Silva G.T.S.T., Santiago E.I., Ribeiro C. (2023). Revisiting Electrocatalytic CO_2_ Reduction in Nonaqueous Media: Promoting CO_2_ Recycling in Organic Molecules by Controlling H_2_ Evolution. Energy Technol..

[2] Fujishima A., Honda K. (1972). Electrochemical Photolysis of Water at a Semiconductor Electrode. Nature.

[3] Ali I., Imanova G., Agayev T., Garibov A., Mansimov Z., Kurniawan T.A., Habila M.A. (2024). Hydrogen production by water spliting using (RaO)_x_(SiO_2_)_y_.H_2_O and gamma radiation. Radiat. Phys. Chem..

[4] Alves G.A.S., Pacholik G., Pollitt S., Wagner T., Rameshan R., Rameshan C., Föttinger K. (2024). Mn-promoted MoS_2_ catalysts for CO_2_ hydrogenation: Enhanced methanol selectivity due to MoS_2_/MnO_x_ interfaces. Catal. Sci. Technol..

[5] Zhai J., Xia Z., Zhou B., Wu H., Xue T., Chen X., Jiao J., Jia S., He M., Han B. (2024). Photo-thermal coupling to enhance CO_2_ hydrogenation toward CH_4_ over Ru/MnO/Mn_3_O_4_. Nat. Commun..

[6] Cigarroa-Mayorga O., Neri E., Calderon H.A., Kisielowski C. (2017). Electron Microscopy of Heterostructure for Solar Energy Recovery: ZnO Nanowires and Co_3_O_4_ Nanoparticles. Microsc. Microanal..

[7] Mendoza-Sánchez A., Cano F.J., Hernández-Rodríguez M., Cigarroa-Mayorga O. (2022). Influence of ZnO Morphology on the Functionalization Efficiency of Nanostructured Arrays with Hemoglobin for CO_2_ Capture. Crystals.

[8] Michael North A., Styring P., Quadrelli E.A., Armstrong K. (2015). Chapter 1—What Is CO_2_? Thermodynamics, Basic Reactions and Physical Chemistry. Carbon Dioxide Utilisation.

[9] He T., Ding W., Cheng X., Cai Y., Zhang Y., Xia H., Wang X., Zhang J., Zhang K., Zhang Q. (2024). Meta-analysis shows the impacts of ecological restoration on greenhouse gas emissions. Nat. Commun..

[10] Schulte R.B., de Arellano J.V.-G., Rutledge-Jonker S., van der Graaf S., Zhang J., van Zanten M.C. (2024). Observational relationships between ammonia, carbon dioxide and water vapor under a wide range of meteorological and turbulent conditions: RITA-2021 campaign. Biogeosciences.

[11] Zhou Y., Li J., Ge W., Liu J., Wu H., Zheng L., Wang X., Qin Y., Zhou J., Wang Y. (2024). Impacts of coal use phase-out in China on the atmospheric environment: (2) public health and global radiative forcing. Atmos. Environ..

[12] World Meteorological Organization (WMO) (2022). The State of Greenhouse Gases in the Atmosphere Based on Global Observations through.

[13] Dong X., Qi D., Chen B., Wu Y., Zheng X., Lin H. (2024). Differential roles of anthropogenic CO_2_ in mediating seasonal amplitudes of ocean acidification metrics over a coastal coral habitat. J. Mar. Syst..

[14] Allen L.H., Vu J.C. (2009). Carbon dioxide and high temperature effects on growth of young orange trees in a humid, subtropical environment. Agric. For. Meteorol..

[15] Rossel R.A.V., Zhang M., Behrens T., Webster R. (2024). A warming climate will make Australian soil a net emitter of atmospheric CO_2_. NPJ Clim. Atmos. Sci..

[16] Le Quéré C., Jackson R.B., Jones M.W., Smith A.J.P., Abernethy S., Andrew R.M., De-Gol A.J., Willis D.R., Shan Y., Canadell J.G. (2020). Temporary reduction in daily global CO_2_ emissions during the COVID-19 forced confinement. Nat. Clim. Change.

[17] Nadiri A., Gündüz V., Adebayo T.S. (2024). The role of financial and trade globalization in enhancing environmental sustainability: Evaluating the effectiveness of carbon taxation and renewable energy in EU member countries. Borsa Istanb. Rev..

[18] Ma D., Xie G., Mao W., Gao J., Yi H., Li D. (2022). Comparison and Improvement of Bioinspired Mobile Algorithms to Trace the Emission Source Based on the Simulation Scenarios. Atmosphere.

[19] Long Y., Li L., Xu T., Wu X., Gao Y., Huang J., He C., Ma T., Ma L., Cheng C. (2021). Hedgehog artificial macrophage with atomic-catalytic centers to combat Drug-resistant bacteria. Nat. Commun..

[20] Saldivar-Ayala D., Ashok A., Cigarroa-Mayorga O., Hernández-Rodríguez Y. (2023). Tuning the plasmon resonance of Au-Ag core-shell nanoparticles: The influence on the visible light emission for inorganic fluorophores application. Colloids Surf. A Physicochem. Eng. Asp..

[21] Hernandez-Rodríguez E., Delgadillo-Moya C. (2020). The ethnobotany of bryophytes in Mexico. Bot. Sci..

[22] Kennedy J.F., Bain de J.F. (1978). Water retention and ion exchange in the leaves of Sphagnum mosses. Plant Physiol..

[23] Pacheco-Cancino P.A., Carrillo-López R.F., Sepulveda-Jauregui A., Somos-Valenzuela M.A. (2024). Sphagnum mosses, the impact of disturbances and anthropogenic management actions on their ecological role in CO_2_ fluxes generated in peatland ecosystems. Glob. Change Biol..

[24] Waraich E.A., Ahmad R., Ashraf M.Y., Saifullah, Ahmad M. (2011). Improving agricultural water use efficiency by nutrient management in crop plants. Acta Agric. Scand. Sect. B—Soil Plant Sci..

[25] Liu Z., Chen Z., Xu X. (2023). Accurate descriptions of molecule-surface interactions for understanding CO_2_ capture by MgO-based sorbents in wet conditions. Carbon Capture Sci. Technol..

[26] Cigarroa-Mayorga O., Gallardo-Hernández S., Talamás-Rohana P. (2021). Tunable Raman scattering enhancement due to self-assembled Au nanoparticles layer on porous AAO: The influence of the alumina support. Appl. Surf. Sci..

[27] Cigarroa-Mayorga O.E., Talamás-Rohana P., Gallardo-Hernández S. (2023). Transmission Electron Microscopy Study on the Process of Gold Nanoporous Film Formation on AAO Substrate by Thermal Treatment. Microsc. Microanal..

[28] Cigarroa-Mayorga O.E. (2021). Tuning the size stability of MnFe_2_O_4_ nanoparticles: Controlling the morphology and tailoring of surface properties under the hydrothermal synthesis for functionalization with myricetin. Ceram. Int..

[29] Ballance S., Kristiansen K., Skogaker N., Tvedt K., Christensen B. (2012). The localisation of pectin in Sphagnum moss leaves and its role in preservation. Carbohydr. Polym..

[30] Ellis L.T., Wilbraham J., Aleffi M., Asthana A.K., Rawat K.K., Gupta D., Sahu V., Katiyar P., Asthana G., Srivastava A. (2018). New national and regional bryophyte records. J. Bryol..

[31] Glime J., Glime J. (2017). Chapter 2-1, Medical Uses: Medical Conditions. Bryophyte Ecology.

[32] Al-Badwy A.H., Khalil A.M., Bashal A.H., Kebeish R. (2023). Polysaccharides from Spirulina platensis (PSP): Promising biostimulants for the green synthesis of silver nanoparticles and their potential application in the treatment of cancer tumors. Microb. Cell Fact..

[33] Addoun N., Boual Z., Delattre C., Chouana T., Gardarin C., Dubessay P., Benaoun F., Addaoud S., El Hadj M.D.O., Michaud P. (2021). Beneficial Health Potential of Algerian Polysaccharides Extracted from *Plantago ciliata* Desf. (Septentrional Sahara) Leaves and Seeds. Appl. Sci..

[34] Yehuda N., Gheber L.A., Kushmaro A., Arad S. (2022). Complexes of Cu–Polysaccharide of a Marine Red Microalga Produce Spikes with Antimicrobial Activity. Mar. Drugs.

[35] Grande-Tovar C.D., Castro J.I., Tenorio D.L., Zapata P.A., Florez-López E., Valencia-Llano C.H. (2023). Chitosan–Polyvinyl Alcohol Nanocomposites for Regenerative Therapy. Polymers.

[36] Buğday N., Ates M.N., Duygulu O., Deng W., Ji X., Altin S., Yaşar S. (2022). ZIF-12-derived N-doped Fe/Co/S/@C nanoparticles as high-performance composite anode electrode materials for lithium-ion batteries. J. Alloys Compd..

[37] Desrues A., De Vito E., Boismain F., Alper J.P., Haon C., Herlin-Boime N., Franger S. (2022). Electrochemical and X-ray Photoelectron Spectroscopic Study of Early SEI Formation and Evolution on Si and Si@C Nanoparticle-Based Electrodes. Materials.

[38] Wang J., Wang J., Wang W., Hu X., Deng Y., Wang H., Wu Y. (2022). The generation of carbon/oxygen double defects in FeP/CoP-N-C enhanced by β particles for photic driving degradation of levofloxacin. Sep. Purif. Technol..

[39] Gbe J.-L.K., Ravi K., Singh M., Neogi S., Grafouté M., Biradar A.V. (2022). Hierarchical porous nitrogen-doped carbon supported MgO as an excellent composite for CO2 capture at atmospheric pressure and conversion to value-added products. J. CO2 Util..

[40] Li Y., Shuai X.-X., Zhang M., Ma F.-Y., Chen J., Qiao J., Chen R.-H., Du L.-Q. (2022). Preparation of ethylenediamine-modified pectin/alginate/Fe3O4 microsphere and its efficient Pb2+ adsorption properties. Int. J. Biol. Macromol..

[41] Khan M.S., Khalid M., Ahmad M.S., Kamal S., Shahid M. (2022). Effect of structural variation on enzymatic activity in tetranuclear (Cu4) clusters with defective cubane core. J. Biomol. Struct. Dyn..

[42] Xiang S., Wang X., Pang Y., Ge C., Xu Y., Chen L., Li S., Wang L. (2022). Porous Au/AAO: A simple and feasible SERSsubstrate for dynamic monitoring and mechanism analysis of DNA oxidation. Appl. Surf. Sci..

[43] Greczynski G., Hultman L. (2022). Referencing to adventitious carbon in X-ray photoelectron spectroscopy: Can differential charging explain C 1s peak shifts?. Appl. Surf. Sci..

[44] Cao X., Zhang D., Cheng X., Xu Q., Zhang L., Huang L., Tu Y., Yu X., Zhang T., Li Y. (2022). Adsorption and Oxidation of CO on Co3O4/Ir(100) Thin Films. J. Phys. Chem. C.

[45] Ramos-Álvarez D., Hernández-Rodríguez Y., Vega-Gómez J., Cigarroa-Mayorga O. (2023). Influence of copper support on the charge transfer enhancement of zinc oxide nanoflakes. Mater. Lett..

[46] Mudgal D., Yadav N., Singh J., Srivastava G.K., Mishra V. (2023). Xanthan gum-based copper nano-magnetite doped carbon aerogel: A promising candidate for environmentally friendly catalytic dye degradation. Int. J. Biol. Macromol..

[47] Yang P.-H., Huang J.-M., Chang Y.-S., Chan C.-T., Hu H.-J. (2023). Fabrication and Characterization of MgO-Based Enzymatic Glucose Biosensors. IEEE Sens. J..

[48] Fazaeli R., Aliyan H., Richeson D., Li Y. (2025). A comparison increasing the photodegradation power of a Ag/g–C3N4/CoNi–LDH nanocomposite: Photocatalytic activity toward water treatment. J. Environ. Sci..

[49] Briggs D.D., Grant J.T. (2003). Surface Analysis by Auger and X-ray Photoelectron Spectroscopies.

[50] Banger K., Yamashita Y., Mori K., Peterson R.L., Leedham T., Rickard J., Sirringhaus H. (2011). Low-temperature, high-performance solution-processed metal oxide thin-film transistors formed by a ‘sol–gel on chip’ process. Nat. Mater..

[51] Liu X., Huo Y.-Q., Yan L.-K., Fan N., Cai K.-Z., Su Z.-M. (2020). Hollow porous MnFe_2_O_4_ sphere grown on elm-money-derived biochar towards energy-saving full water electrolysis. Chem. Eur. J..

[52] Sharmila A., Selvaraj C.I. (2024). Phyto-synthesized MgO nanoparticles using Scutia myrtina Kurz extract: Promising insights into photocatalytic degradation, antioxidant potential, cytotoxicity and toxicity assessment. J. Mol. Struct..

[53] Mohan T., Kumar J., Roy I. (2023). Iron selenide nanorods for light-activated anticancer and catalytic applications. New J. Chem..

[54] Lin L., Han S., Meng F., Li J., Chen K., Hu E., Jiang J. (2024). The influence of pore size and pore structure of silica-based material on the amine-modified adsorbent for CO_2_ capture. Sep. Purif. Technol..

[55] Yang J., Gao D., Zhang H., Yi Q. (2024). Construction of ZIF-8 and amino functionalized porous ionic liquids for efficient CO_2_ capture. Fuel.

[56] Aliyu M., Yusuf B.O., Abdullahi A.S., Bakare A.I., Umar M., Hakeem A.S., Ganiyu S.A. (2024). Ti_2_C-MXene/activated carbon nanocomposite for efficient CO_2_ capture: Insights into thermodynamics properties. Sep. Purif. Technol..

[57] Yuan H., Li P., Sun X., Cen D., Luo D., Yan X., Lei G., Zheng W., Hu Z., Yang R.T. (2024). Amine-grafted on boron modified SBA-15 for direct air capture of CO_2_. Sep. Purif. Technol..

[58] Zhao D., Liu H., Huang Q., Yu L., He Z., Lu H., Li Q. (2024). Tetraethylenepentamine-modified Cu_2_(OH)PO_4_ for efficient CO_2_ capture. Sep. Purif. Technol..

[59] An M., Guo T., Guo Q. (2024). Facile preparation of coal-based ultramicroporous carbon microspheres for selective CO_2_ capture. Carbon Resour. Convers..

